# An effective biphase system accelerates hesperidinase-catalyzed conversion of rutin to isoquercitrin

**DOI:** 10.1038/srep08682

**Published:** 2015-03-03

**Authors:** Jun Wang, An Gong, Cai-Feng Yang, Qi Bao, Xin-Yi Shi, Bei-Bei Han, Xiang-Yang Wu, Fu-An Wu

**Affiliations:** 1 School of Biotechnology, Jiangsu University of Science and Technology, Zhenjiang. 212018, P R China; 2 Sericultural Research Institute, Chinese Academy of Agricultural Sciences, Zhenjiang. 212018, P R China; 3 School of the Environment and Safety Engineering, Jiangsu University, Zhenjiang. 212013, P R China

## Abstract

Isoquercitrin is a rare, natural ingredient with several biological activities that is a key precursor for the synthesis of enzymatically modified isoquercitrin (EMIQ). The enzymatic production of isoquercitrin from rutin catalyzed by hesperidinase is feasible; however, the bioprocess is hindered by low substrate concentration and a long reaction time. Thus, a novel biphase system consisting of [Bmim][BF_4_]:glycine-sodium hydroxide (pH 9) (10:90, v/v) and glyceryl triacetate (1:1, v/v) was initially established for isoquercitrin production. The biotransformation product was identified using liquid chromatography-mass spectrometry, and the bonding mechanism of the enzyme and substrate was inferred using circular dichroism spectra and kinetic parameters. The highest rutin conversion of 99.5% and isoquercitrin yield of 93.9% were obtained after 3 h. The reaction route is environmentally benign and mild, and the biphase system could be reused. The substrate concentration was increased 2.6-fold, the reaction time was reduced to three tenths the original time. The three-dimensional structure of hesperidinase was changed in the biphase system, which *α*-helix and random content were reduced and *β*-sheet content was increased. Thus, the developed biphase system can effectively strengthen the hesperidinase-catalyzed synthesis of isoquercitrin with high yield.

Isoquercitrin (quercetin-3-O-*β*-D-glucoside), a natural, rare ingredient with several biological activities, has attracted increasing attention. Isoquercitrin is generally used as an antithrombotic drug to treat myocardial ischemia, cerebral hypoxia and ischemic disease because of its non-oxidizable^1^, anti-inflammatory^2^, anti-depressant, hypotensive, hypolipidemic^3^, anti-mutagenesis^4^, and anti-virus properties as well as other pharmacological effects. In addition, isoquercitrin is a key precursor for the biosynthesis of enzymatically modified isoquercitrin (EMIQ), which was recently approved as a multiple food additive^5^,^6^. However, isoquercitrin rarely exists in nature^7^ and the extraction yield of isoquercitrin is extremely low by using traditional extraction methods, which results in high production costs^8^. Isoquercitrin is a derivative of rutin that lacks a rhamnose moiety in its structure. Recently, several methods for the transformation of rutin to isoquercitrin have been investigated, including acid hydrolysis^9^, heating^10^, microbial transformation^11^, and enzymatic transformation techniques^9^. Due to its significant economic benefits and ecological acceptability compared to solvent extraction from natural sources and chemical synthesis^12^, there are increasing reports indicating that isoquercitrin can be efficiently synthesized from rutin using enzymatic hydrolysis process under suitable reaction conditions^13^.

In recent years, the enzymatic production of isoquercitrin by selective transformation of rutin in buffer systems has been successively established. Hesperidinase (hesperidin-*α*-1,6-rhamnosidase, EC 3.2.1.40) is an excellent commercially available biocatalyst for the biotransformation of rutin to isoquercitrin with high selectivity^14^. Compared with crude and recombinant *α*-L-rhamnosidases, hesperidinase was proposed as a more technically feasible catalyst through the control of pH instead of temperature^14^,^15^. Subsequently, a buffer medium containing a specific proportion of the ionic liquid (IL) [Bmim][BF_4_] was developed as a co-solvent system^9^. [Bmim][BF_4_]-glycine-sodium hydroxide buffer (pH 9) (10:90, v/v) was determined to be the optimal medium, which effectively improved rutin solubility and hesperidinase catalytic efficiency. These results indicated that the IL can effectively enhance the selective synthesis of isoquercitrin and the reaction process is simple and eco-friendly^10^. When the substrate concentration of rutin was increased by 9.8-fold, the reaction time was reduced from 30 h to 10 h, the conversion of rutin was improved to 93.4% (1.7-fold), and the isoquercitrin yield was enhanced to 91.4% (2.3-fold). However, further industrial application of the hesperidinase-catalyzed transformation in the co-solvent system remains limited by low substrate solubility, slow catalytic efficiency and long reaction time. Therefore, it is imperative to explore more efficient processes for the hesperidinase-catalyzed synthesis of isoquercitrin from rutin via rhamnose hydrolysis.

Biphasic systems have long attracted attention. Biphasic extraction has been used to extract isoquercitrin from a mixture during the reaction process. In this type of system, the catalyst is located in the mobile phase. It is generally believed that the reaction occurs in the aqueous phase or at the phase interface^16^,^17^. After the completion of the reaction, a simple phase-separating operation could allow the separation of the catalyst and product^18^. For the hesperidinase-catalyzed synthesis of isoquercitrin from rutin, there is no doubt that this type of extraction is one of the best and simple methods to prepare isoquercitrin in an IL-buffer co-solvent system. In our follow-up study, we determined that glyceryl triacetate is a more suitable extracting agent and the solvent can be recycled and reused. According to dissolve balance principle, isoquercitrin molecules in the enzymatic reactants could be quickly enter the extraction phase, reducing the target product, which shifts the equilibrium reaction in the desired direction^19^. Therefore, if a biphase system was applied in the hesperidinase-catalyzed synthesis of isoquercitrin from rutin, it would accelerate the biocatalytic progress and also significantly improve isoquercitrin yield. Based on the above, the investigated enzymatic reaction of isoquercitrin biosynthesis using selective conversion of rutin catalyzed by hesperidinase was shown in Fig. 1. However, to the best of our knowledge, no report has been published concerning the application of a biphase system for the hesperidinase-catalyzed synthesis of isoquercitrin from rutin.

In general, enzymes are the globular proteins whose catalytic activity depends on native configuration of their active-sites. Different systems may cause various alterations to enzyme secondary structure, leading to better exposure of active sites of enzyme^20^. Fluorescence spectroscopy is a useful method for analyzing structure alterations in proteins because the intrinsic fluorescence of aromatic amino acid residues is susceptible to the polarity of microenvironments during the transition^21^. Consequently, to better understand the effect of different systems (i. e., aqueous, co-solvent, and biphase systems) on the activity of hesperidinase and its secondary structure, hesperidinase conformation changes were initially evaluated by measuring the intrinsic fluorescence and CD spectra in the presence and absence of other substances^22^,^23^,^24^.

The purpose of this study was to select the optimal reaction conditions for isoquercitrin enzymatic synthesis in a [Bmim][BF_4_]-buffer/glyceryl triacetate (1:1, v/v) system. Five factors including the pH value of the buffer, temperature, substrate concentration, enzyme concentration, and volume ratio of two phases were investigated. In addition, the apparent kinetic parameter *V*_m_/*K*_m_ was also determined and the bonding mechanism between the enzyme and substrate was integrated using circular dichroism (CD).

## Results and Discussion

### Effect of pH

**Figure 2:**
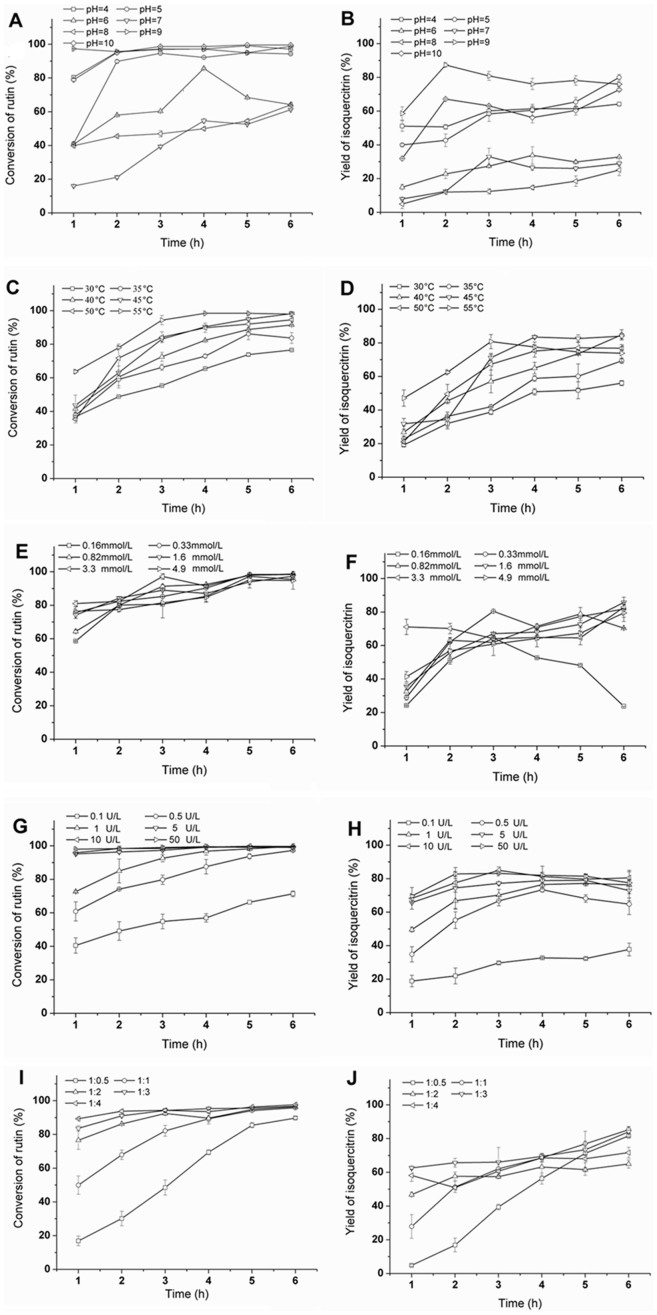
The effect of reaction conditions on enzymatic synthesis of isoquercitrin from rutin during the enzymatic transformation of rutin using a biphase system. Reaction conditions: the effect of pH on rutin conversion (A) and isoquercitrin yield (B) using the [Bmim][BF_4_]-buffer/glyceryl triacetate (1:1, v/v) system in the presence of hesperidinase (10 U/L) at 40°C and 180 rpm for 6 h; the rutin concentration was 0.33 mmol/L, and the volume ratio of substrate and glyceryl triacetate was 1:1. The effect of temperature on rutin conversion (C) and isoquercitrin yield (D) using the [Bmim][BF_4_]-buffer/glyceryl triacetate (1:1, v/v) system at 180 rpm for 6 h; the rutin concentration was 0.33 mmol/L, and the volume ratio of substrate and glyceryl triacetate was 1:1. The effect of substrate concentration on rutin conversion (E) and isoquercitrin yield (F) using the [Bmim][BF_4_]-buffer/glyceryl triacetate (1:1, v/v) system in the presence of hesperidinase (10 U/L) at 180 rpm for 6 h; and the volume ratio of substrate and glyceryl triacetate was 1:1. The effect of enzyme concentration on rutin conversion (G) and isoquercitrin yield (H) using the [Bmim][BF_4_]-buffer/glyceryl triacetate (1:1, v/v) system in the presence of rutin concentration (0.33 mmol/L) at 40°C and 180 rpm for 6 h; the volume ratio of substrate and glyceryl triacetate was 1:1. The effect of the volume ratio of the two phases on rutin conversion (I) and isoquercitrin yield (J) using the [Bmim][BF_4_]-buffer/glyceryl triacetate (1:1, v/v) system in the presence of hesperidinase (10 U/L) at 45°C 180 rpm for 6 h; the substrate concentration was 3.3 mmol/L.

Figs. 2A and 2B show the effects of pH on rutin conversion and isoquercitrin yield during the hesperidinase-catalyzed transformation of rutin in a biphase system, respectively. [Bmim][BF_4_] was used as a co-solvent and the rutin solution was had a pH range of 4–10. Fig. 2A shows that the buffer solution in the [Bmim][BF_4_]-buffer/glyceryl triacetate (1:1, v/v) system at pH 4, 5, 9 and 10 have higher enzymatic transformation rates for rutin with conversions of 99.2%, 95.0%, 98.8% and 99.6%, respectively. However, Fig. 2B shows that when the pH value was simply adjusted to 9, the enzymatic biotransformation of rutin reached the highest yield of isoquercitrin, 87.4%. Therefore, the optimal buffer solution pH value in the [Bmim][BF_4_]-buffer/glyceryl triacetate (1:1, v/v) system was 9. In addition, pH 5 was an optimal pH value in the biphase system for high selectivity of the bioconversion of rutin to isoquercitrin. However, rutin solubility in pH 5 buffer solutions is lower than in pH 9 buffer solutions^9^, therefore, it is unsuitable for industrial production.

The pH value has an important impact on the solubility of enzymes and substrates, which affects enzyme conformation, catalytic activity and stability^25^,^26^,^27^ as well as rutin conversion and isoquercitrin production. Increasing the available substrate that can interact with the enzyme improves the reaction probability and efficiency. Preliminary results determined that pH 6 is the optimal pH value of hesperidinase for isoquercitrin production in an aqueous buffer medium. The *α*-L-rhamnosidase and *β*-D-glucosidase enzymes do not have any activity at pH 8. However, pH 9 was the optimal pH value of the buffer solution in the biphase system. This is because the dissociation constants (p*K*_a_) of the different acid-base couples vary in the same media, establishing an acidity scale in buffer-[Bmim][BF_4_] mixtures after the addition of IL to the buffer solution^28^. Based on the above results in the biphase medium, the nature of the IL co-solvents considerably alters hesperidinase catalytic activity, resulting in significant changes to all respects of the reaction parameters.

### Effect of temperature

As shown in Figs. 2C and 2D, the effect of temperature on rutin conversion and isoquercitrin yield in the hesperidinase-catalyzed transformation of rutin in a biphase system were investigated at 30–55°C. The concentration of aqueous rutin solution was 3.3 mmol/L and pH was 9.0. The results indicated that the optimal temperature range for the selective biotransformation of rutin to isoquercitrin was 40 to 50°C. The highest isoquercitrin yield occurred at 40°C. Additionally, the hesperidinase *α*-L-rhamnosidase activity was slightly reduced above 50°C.

As the temperature further increased, the selectivity of isoquercitrin production was reduced via rutin hydrolysis in the [Bmim][BF_4_]-buffer/glyceryl triacetate (1:1, v/v) system. This may be attributed to the hesperidinese, a complex enzyme containing *α*-rhamnosidase and *β*-glucosidase, however, *α*-rhamnosidase primarily catalyzes the hydrolysis reaction^29^. Additionally, above 50°C there may be sufficient energy to disrupt some intra-molecular attractions between polar groups as well as disrupt the hydrophobic forces between non-polar groups within the protein structure. When those forces are altered by external factors, they alter the tertiary and secondary enzyme protein structures, which will alter the conformation of the enzyme active site^30^. Therefore, the optimum enzymatic reaction temperature should be 45°C for industrial application. Compared with the [Bmim][BF_4_]-buffer system, the reaction temperature increased 5°C in the biphase system, which is favorable for industrial production.

### Effect of substrate concentration

Figs. 2E and 2F show the effects of substrate concentration (0.16–4.9 mmol/L) on rutin conversion and isoquercitrin yield in the hesperidinase-catalyzed transformation of rutin in the [Bmim][BF_4_]-buffer/glyceryl triacetate (1:1, v/v) system. The reactions were incubated in aqueous rutin solution with pH 9 at 45°C. The reaction was sampled every hour for six hours. The results indicated that when the rutin substrate concentration increased from 0.16 to 4.9 mmol/L, the isoquercitrin yield increased from 24.2% to 71.1% after 1 h, with the highest yield at 3.3 mmol/L rutin. The isoquercitrin yield increased from approximately 23.7% to 85.7% after 6 h with the highest yield at 3.3 mmol/L of rutin. This is because the enzymatic reaction rate may be induced by higher concentrations of substrate and hesperidinase in the aqueous phase, leading to increased hesperidinase concentrations at the interface within the biphase system. This effect would decrease hesperidinase activity because it would be surrounded by excess organic solvent^31^. In addition, the rutin substrate concentration in the biphase system was 2.6-fold higher than that in the [Bmim][BF4]-buffer (10:90, v/v) system^9^.

Substrate inhibition and product degradation are possible reasons for the decrease in isoquercitrin conversion when the rutin concentration was increased^32^. Isoquercitrin yields at rutin concentrations (0.16, 0.33, 0.82 and 4.9 mmol/L) were relatively lower than those at rutin concentrations (1.6 and 3.3 mmol/L). The results indicated that the substrate and product both were unstable in this situation, because they are natural flavonoids with highly antioxidant activity. A small number of isoquercitrin could be derived to other compounds, and some rutin could be hydrolyzed to other substances, such as astragalin, hyperoside and taxifolin^33^,^34^. In addition, quercetin was most probably generated in the biphasic reaction as the reaction was executed^35^. Therefore, isoquercitrin yield probably demonstrated a strange tendency, which shows a strongly different behavior. In general, when the rutin concentration was 1.6 mmol/L, a higher isoquercitrin yield should be obtained. However, if isoquercitrin is abundantly produced and the substrate concentration is appropriately increased, the yield and efficiency should be greatly improved. Therefore, the putative optimal substrate concentration for isoquercitrin production is 3.3 mmol/L.

### Effect of enzymatic activity

Figs. 2G and 2H show the effects of the enzymatic activity (0.1–50 U/L) on rutin conversion and isoquercitrin yield in the hesperidinase-catalyzed transformation of rutin in [Bmim][BF_4_]-buffer/glyceryl triacetate (1:1, v/v) system. When the enzyme concentration was increased from 0.1 U/L to 50 U/L, the isoquercitrin yield and rutin conversion both generally increased. Isoquercitrin yield began to maintain a relatively stable value in (80 ± 5)% at 3 h, and then decreased with increasing reaction time. The results indicated that under certain conditions of temperature and pH, the reaction rates increases with increasing amount of enzyme concentration. After 3 h, a longer reaction time caused such a loss of isoquercitrin. To reduce costs of enzymatic reaction in the biphase system, the optimal enzyme amounts was selected as 10 U/L. The maximum isoquercitrin yield, 85.0%, was obtained in the biphase system after 3 h, which was 3.3 times faster than the [Bmim][BF_4_]-buffer (10:90, v/v) system (10 h). In addition, as shown in Table 1, the hesperidinase concentration was decreased from 100 U/L (10 g/L) to 10 U/L (1 g/L); tremendously improving efficiency and immensely reducing costs.

Because hesperidinase in a biphase system is a free enzyme, its secondary structure is exposed, which is favorable for protein and rutin interaction during enzymatic hydrolysis. When the hesperidinase active site is obscured by adjacent sites^36^,^37^,^38^, it cannot sufficiently interact with substrates, preventing the catalytic activity of the enzyme molecules and reducing the catalytic efficiency of hesperidinase.

### Effect of the volume ratio of two phases

Figs. 2I and 2J show the effects of the volume ratio of the two phases on rutin conversion and isoquercitrin yield during the hesperidinase-catalyzed transformation of rutin in the [Bmim][BF_4_]-buffer/glyceryl triacetate (1:1, v/v) system. Five different phase proportions were selected: 1:0.5, 1:1, 1:2, 1:3 and 1:4. As the reaction progressed, the rutin conversion rate gradually increased and isoquercitrin yield gradually improved to 85.2% after 6 h. As the volume ratio of glyceryl triacetate and substrate increased, the time required for the reaction decreased. However, when the volume ratio of the two phases was 1:1, the final isoquercitrin yield was higher than a volume ratio of 1:4. In the previous experiment, this extraction method in the reaction rarely occurred. During the enzymatic reaction, isoquercitrin was extracted by glyceryl triacetate, shifting the chemical equilibrium to the relative right and improving the reaction rate.

The reaction catalyzed by hesperidinase occurs in the aqueous bulk phase, not at the organic interface. Thus, the mass-transfer rate of the extraction agent from the organic phase to the aqueous phase may also influence the reaction rate^39^. No significant difference was observed as the volume ratios were examined, suggesting that the phase volume ratio may not be a sensitive parameter for affecting enzyme stability in a biphase system^40^. If excess organic solvent is added, the product generates some amount of pollution. In addition, excess organic solvent causes waste according to the principles of green chemistry.

### The reaction time and isoquercitrin extracted repeatedly

**Figure 3:**
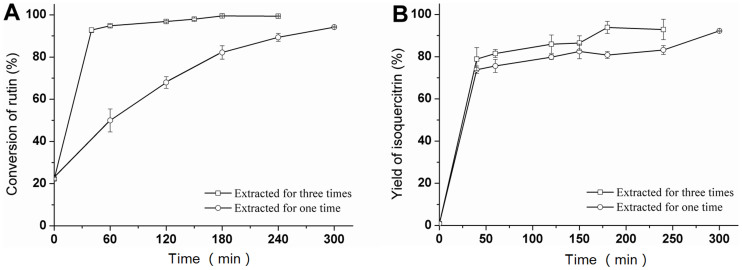
Rutin conversion (A) and isoquercitrin yield (B) during the enzymatic transformation of rutin for the study of dynamics using a biphase system. The conditions of the system were: a [Bmim][BF_4_]-buffer/glyceryl triacetate (1:1, v/v) system and the presence of hesperidinase (10 U/L) at 45°C 180 rpm; the substrate concentration was 3.2 mmol/L.

The effect of reaction time on enzymatic synthesis of isoquercitrin was investigated over a range of 0.25–5 h. Fig. 3A shows the rutin conversion during the enzymatic reaction in the [Bmim][BF_4_]-buffer/glyceryl triacetate (1:1, v/v) system. The rutin conversion was 96.9% and 99.5% after 1 h and 3.5 h, respectively. Fig. 3B shows the isoquercitrin yield during the enzymatic reaction in a biphase system. With an increased reaction time, the isoquercitrin yield increased linearly during the early stage of enzymatic hydrolysis. However, the isoquercitrin yield was only 75.6% and 83.1% after 1 h and 3.5 h, respectively.

An additional experiment was conducted with a 0–4 h reaction time to inspect the isoquercitrin yield. After terminating the reaction, the organic phase was removed. Fresh organic phase (glyceryl triacetate) was added and extracted for 5 min ultrasonically; then, the organic phase was removed. After extracting 5 times using this method, the rutin conversion and isoquercitrin yield increased to 99.5% and 93.9% after 3 h, respectively. Therefore, 3 h was determined to be a suitable reaction time.

Table 1 also shows the optimized time and temperature using a [Bmim][BF_4_]-buffer/glyceryl triacetate (1:1, v/v) system in comparison with the previous experiments. Using the biphase system, the reaction time was reduced from 10 h to 3 h^9^.

### The reaction rate and the calculation of apparent Michaelis constant in a biphase system

**Figure 4:**
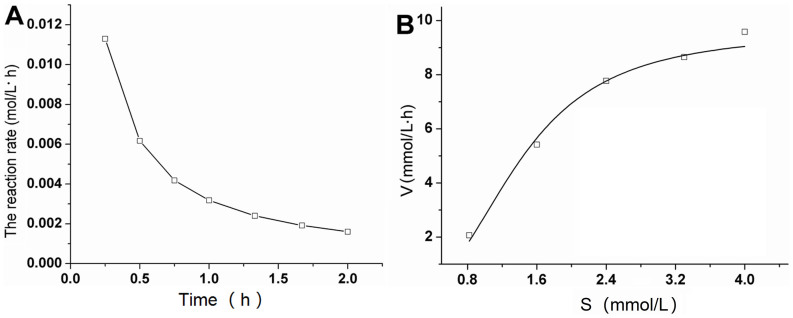
The effect of substrate concentration on the initial reaction rate during the hesperidinase-catalyzed transformation of rutin to isoquercitrin in a biphase system. The concentrations of rutin and hesperidinase were 3.3 mmol/L and 10 U/L, respectively. All experiments were performed at 45°C and 180 rpm in the [Bmim][BF_4_]-buffer/glyceryl triacetate (1:1, v/v) system. (A) The enzymatic reaction rate under optimal conditions in a biphase system; (B) The kinetics of biphase system of the initial reaction rate using non-linear regression methods for the determination of the apparent *K*_m_-value during the hesperidinase-catalyzed transformation of rutin to isoquercitrin.

Fig. 4A shows the effect of substrate concentrations on the initial reaction rate during the hesperidinase-catalyzed conversion of rutin for the production of isoquercitrin in a biphase system. The rutin reaction rate was rapid during the initial reaction in the biphase system. As the reaction continued, the rutin reaction rate gradually decreased until 1.5 h. After a 2 h reaction, less rutin remained in the system and the reaction rate was relatively slower.

Fig. 4B illustrates the apparent *K*_m_ value and *V*_m_ by using non-linear regression methods in a biphase system. The apparent Michaelis constant and the maximum velocity of the reaction (*V*_m_) can be calculated using this plot. The reaction rate of this enzymatic reaction increased when the substrate concentration was raised or the reaction temperature was within a suitable range^41^. In addition, affinity of enzymes toward the substrates could be evaluated by apparent Michaelis constant *K*_m_, and the smaller apparent Michaelis constant indicates the higher affinity^42^. In the developed biphase system, the initial rate of isoquercitrin production was 0.017 mol/L·h, the apparent kinetic parameter *V*_m_*/K*_m_ (*V*_m_ = 9.6 mmol/L·h, *K*_m_ = 1.4 mmol/L) was 6.9 h^−1^, which was much less than that in a monophase system (1531.4 h^−1^)^9^. But in this experiment, the affinity of hesperidinase toward rutin in a biphase system was far lower than that in a monophase system. This maybe because of the existing of the reaction interface in the biphase system, most of the substrates transferred to the interface and the enzyme molecular could not contact rutin.

Table 1 shows the comparative results of the enzymatic transformation of rutin to isoquercitrin in a biphase [Bmim][BF_4_]-buffer/glyceryl triacetate (1:1, v/v) system and [Bmim][BF_4_]-buffer (10:90, v/v) system. Compared with the co-solvent system, the biphase system had significant advantages in both reaction parameters and catalytic results with high yield and selectivity. According to the enzymatic reaction parameters, the substrate concentration of rutin in the biphase system was 2.6-fold higher than the [Bmim][BF_4_]-buffer (10:90, v/v) system and the apparent kinetic parameter was reduced to 5% the original value in [Bmim][BF_4_]-buffer (10:90, v/v) system. Regarding the catalytic results, the rutin conversion and isoquercitrin yield in the biphase system were 1.07-fold and 1.03-fold higher, respectively, than in the [Bmim][BF_4_]-buffer (10:90, v/v) system.

This reaction route is environmentally benign and mild, the enzyme and the biphase system contain [Bmim][BF_4_]:glycine-sodium hydroxide (pH 9) and glyceryl triacetate could be reused. Importantly, glyceryl triacetate is nontoxic and clean. Isoquercitrin extracted by glyceryl triacetate also could be used as a safe and harmless food additive, or a precursor for the synthesis of EMIQ. Thus, the biphase system can effectively strengthen the hesperidinase-catalyzed synthesis of isoquercitrin from rutin with high yield.

### Effect of different systems on the structure of hesperidinase

**Figure 5:**
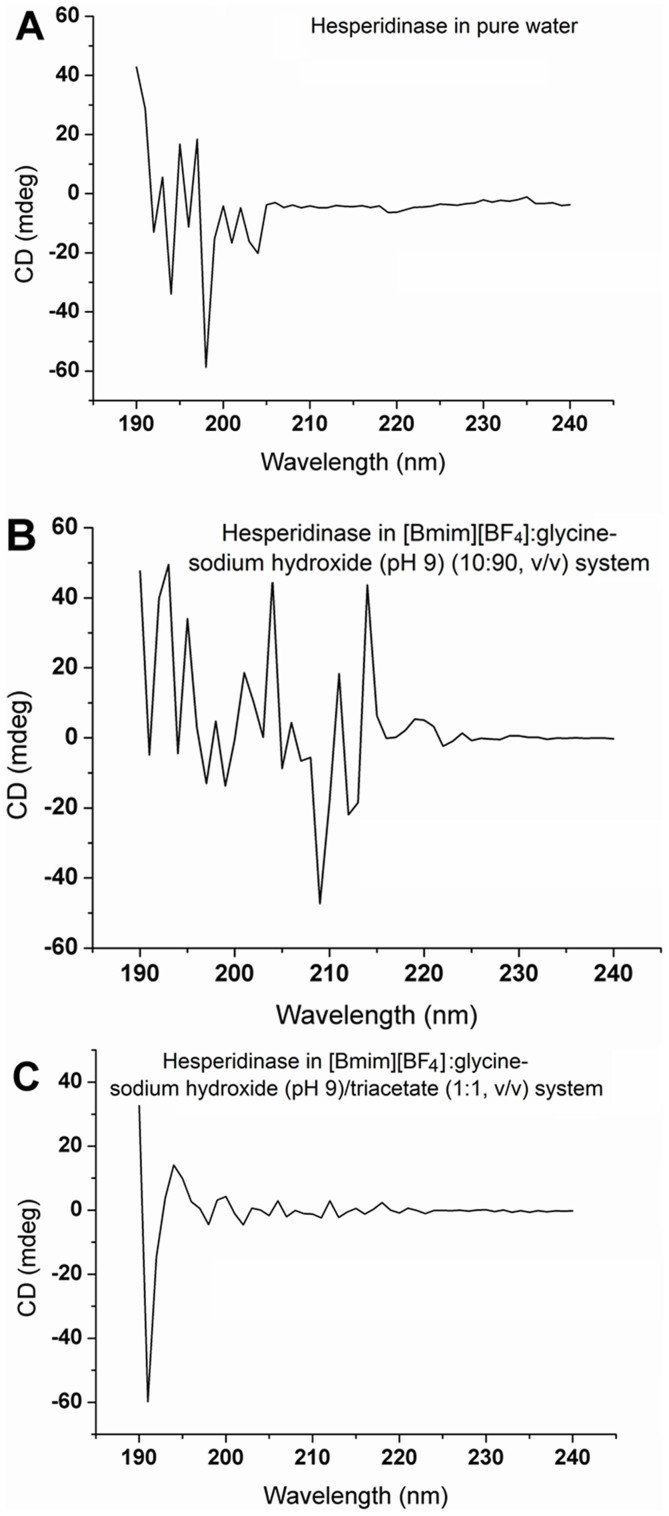
CD spectra of hesperidinase in pure water (A), [Bmim][BF_4_]:glycine-sodium hydroxide (pH 9) (10:90,v/v) (B) and [Bmim][BF_4_]:glycine-sodium hydroxide (pH 9) (10:90,v/v)/glyceryl triacetate (1:1,v/v) (C). The hesperidinase concentration was 0.18 U/L. All samples were placed in a water thermostat at 25.0°C prior to the experiments and were detected before the reaction with the following conditions: 45°C, 180 rpm for 6 h in a shaker.

The analysis of the hesperidinase CD spectra in three systems (including aqueous, co-solvent, and biphase systems) are shown in Fig. 5 and are summarized in Table 2. The content of *α*-helix, *β*-sheet, turn and random of hesperidinase was calculated to understand the association between enzyme activity and secondary structure. As shown in Table 2, the fraction of *α*-helix decreased in the [Bmim][BF_4_]-buffer/glyceryl triacetate (1:1, v/v) system. A decrease in the *α*-helical fractions in hesperidinase, resulting from accelerated extracted mass transfer, may be attributed to turbulence^43^ and can induce structural transformations that may affect the enzyme active site. Table 2 shows that in the biphase system, the contents of *α*-helix and random in the [Bmim][BF_4_]-buffer/glyceryl triacetate (1:1, v/v) system were decreased by 9.6% and 30.1%, and those in the [Bmim][BF_4_]-buffer (10:90, v/v) system were decreased by 6.5% and 26.8%, respectively, compared with untreated hesperidinase. The *β*-sheet content in the [Bmim][BF_4_]-buffer/glyceryl triacetate (1:1, v/v) system was increased by 35.1%. The contents of *α*-helix and random in the [Bmim][BF_4_]-buffer/glyceryl triacetate (1:1, v/v) system were decreased by 3.1% and 3.3% compared with the [Bmim][BF_4_]-buffer (10:90, v/v) system.

These alterations increased the uniformity and flexibility of hesperidinase, which enhances its activity and improves catalytic efficiency^20^. The decreased proportion of *α*-helix and random and the increased proportion of *β*-sheet in hesperidinase is more conducive for isoquercitrin production. Wang et al.^44^ reported similar results for changes in the composition of cellulase structure with an 8.85% decrease in *α*-helix content. Combined with the results of the previous experiments, the small proportion of *α*-helix in the reaction system was more favorable for the reaction.

In conclusion, the extraction agent glyceryl triacetate was successfully applied in a biphase system for the selective biotransformation of rutin for isoquercitrin production. The rutin conversion and isoquercitrin yield in the [Bmim][BF_4_]-buffer/glyceryl triacetate (1:1, v/v) system were 99.5% and 93.9%, respectively. Compared with the co-solvent system, the developed biphase system increased the substrate concentration 2.6-fold, decreased the reaction time to three tenths the original time, increased the reaction temperature from 40°C to 45°C, and hesperidinase had a higher substrate affinity to rutin in a biphase system. The results suggest that the developed biphase system could enhance the hesperidinase-catalyzed synthesis of isoquercitrin from rutin with high yield and selectivity.

## Methods

### Synthesis of isoquercitrin in a biphase system

In this study, disodium hydrogen phosphate-citrate buffer (pH 4–8) and glycine-sodium hydroxide buffer (pH 9–10) were used in a biphase system. The system contains: 720 *μ*L rutin solution, 100 *μ*L IL, 180 *μ*L hesperidinase solution and 1 mL glyceryl triacetate. The hesperidinase (contains *α*-L-rhamnosidase and *β*-D-glucosidase activities, ≥10 units/g solid) produced by *Penicillium* was purchased from Sigma Co. (St. Louis, MO, USA). All enzymatic reactions were performed in a temperature controlled incubator shaker. In a typical experiment, rutin buffer solution was added with the IL to a 10 mL centrifuge tube. The reaction was initiated by adding hesperidinase and glyceryl triacetate buffered solution and the mixtures were incubated for different amounts of time at various pH values, IL concentrations, substrate concentrations, enzyme concentrations, reaction times, and the volume ratio of two phases with diffferent amount of glyceryl triacetate. Glyceryl triacetate is an excellent solvent to extract isoquercitrin from aqueous phase. The partition coefficients of the reactant (rutin) and product (isoquercitrin) were 1.6 and 0.25, respectively, within this biphasic system, by which the method was described in supplementary material. During these experiments, the other conditions were fixed in a temperature-controlled heating water bath. The mixture was shaken at 180 rpm at different temperatures and sampled for every hour for six hours. The crude hydrolysis products of rutin were then centrifuged at 10,000 rpm for 10 min. Next, 500 *μ*L supernatant solutions and 500 *μ*L subnatant solutions were filtered through a 0.45 *μ*m filter prior injection into HPLC.

### Analysis of transformed products by HPLC and LC-MS

HPLC quantitative analyses were performed using a HITACHI Pump L-7100 with a UV–VIS Detector (PLC-2, Biochem. Jinda. Ltd., Shanghai, China) and an N-2000 workstation (Hangzhou Mingtong S&T Ltd., Hangzhou, China). An Alltima C_18_ column (250 mm × 4.6 mm, i.d.; 5 μm, W. R. Grace & Co., Deerfield, IL, USA) was used, and the column was maintained at 30°C. The separation and determination of rutin and isoquercitrin was performed using an HPLC/UV method on an Alltima C_18_ column with a mobile phase consisting of acetonitrile: 0.02% phosphoric acid solution (20:80, v/v) at a flow rate of 1.0 mL/min. Rutin and isoquercitrin, were all detected at 360 nm^6^. All samples were assayed in triplicate.

Rutin conversion and isoquercitrin yield of the hesperidinase-catalyzed isoquercitrin synthesis were calculated as follows, respectively.
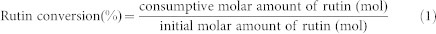

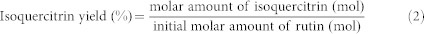


LC-MS was performed on a Thermo Fisher system. The LC equipment comprised a Finnigan MAT Spectra System P4000 pump, an autosampler with a 50 *μ*L loop, a UV6000LP diode array detector and a Finnigan AQA mass spectrometer. LC separation was performed on an Alltima C_18_ column (250 mm × 4.6 mm, i.d.; 5 *μ*m, W. R. Grace & Co., Deerfield, IL, USA). The mobile phases consisted of 0.1% formic acid in water (A) and 0.1% formic acid in acetonitrile (B). Separation was performed with the following conditions: 0–35 min, 6–100% B; 35–40 min, back to 6% B. The column was equilibrated for 15 min prior to each analysis. The wavelength range of the PAD was 200 to 400 nm. The flow rate was 1.0 mL/min for LC, and the column remained at 40°C during PAD detection. Electrospray ionization (ESI) was performed using nitrogen to assist nebulization (1.0 mL/min flow rate). Selected ion monitoring (SIM) in negative ion mode with 1.6 KV capillary voltage was used and the temperature of the curved desolvation line (CDL) and heat block were both set at 200°C. The data were processed using Xcalibur 1.2 software. The intense peaks at m/z 463.17 in the ESI-MS spectra under negative ion mode corresponded to the deprotonated [M–H]^−^ ions of isoquercitrin^9^.

### Determination of kinetic parameters and apparent michaelis constant

Kinetic parameters were conventionally determined from the initial rate data and non-linear regression to the corresponding rate equations. The concentrations of rutin and hesperidinase solution were 3.3 mmol/L and 10 U/L, respectively. All experiments were performed in a [Bmim][BF_4_]-buffer/glyceryl triacetate (1:1, v/v) system at 45°C and 180 rpm in a shaker. All samples were assayed in triplicate and were obtained at 0, 10, 20, 30, 40, 50, 60, 80, 100, 120, 150, 180, 210 and 240 min. To attain equilibrium, the samples were placed in a refrigerator at −20°C before HPLC detection. The reaction rate of different time points were calculated using the HPLC data.

The apparent Michaelis constant, *K*_m_, is the most important constants in the study of enzymatic reaction kinetics in a biphase system. Different substrate concentrations of rutin solution (0.82, 1.6, 2.4, 3.3 and 4.0 mmol/L) were prepared to obtain the initial rate of isoquercitrin production in a [Bmim][BF_4_]-buffer/glyceryl triacetate (1:1, v/v) system with hesperdinase activity of 10 U/L at 45°C, 180 rpm/min in a shaker. Every sample was obtained at 15 min to determine the initial conversion rate. Apparent *K*_m_ value and *V*_m_ were calculated by using non-linear regression methods.

### Multiple interfacial isoquercitrin extraction methods

Because an interface exists in a biphase system, there may be an amount of isoquercitrin adsorbing at the interface^45^. Isoquercitrin can be easily extracted using organic phase glyceryl triacetate; therefore, repeated extractions can accelerate the reaction time. The reaction system was a [Bmim][BF_4_]-buffer/glyceryl triacetate (1:1, v/v) system with the following conditions: hesperdinase (10 g/L), rutin (3.3 mmol/L), 45°C, 180 rpm in a shaker. After a one hour reaction time, the organic phase was removed and fresh organic phase was added, repeated five times. Samples were placed in a refrigerator at −20°C before detecting by HPLC. The concentration of each sample was added to determine the total isoquercitrin concentration.

### CD spectroscopic assay

CD measurement was carried out with Jasco-810 spectropolarimeter (Japan) in Yangzhou University. The CD spectra were measured in 190–300 nm at room temperature using a 0.1 cm quartz cuvette. The scanning speed was 100 nm*/*min; bandwidth was 1 nm; spectral response was 0.1 nm. The data points were taken on an average of four times. The *α*-helix and *β*-sheet structure contents in the protein were calculated using the software provided by the manufacturer (Jasco-810 Analytical Manager System) corresponding to standard substances.

All the experiments were carried out at 25°C. To attain equilibrium, the reaction samples were placed in a water thermostat at 25°C before the experiments.

### Statistics analysis

Triplicate experiments were performed for each parameter investigated. Standard deviations were calculated to verify the reliability of the results. The differences in mean values were evaluated using the analysis of variance (ANOVA) method. Significance was determined at a 95% level of probability.

## Author Contributions

J.W. conceived the idea and designed the experiments. A.G. prepared the samples and performed characterization with the assistance from C.Y., Q.B., X.S. and B.H. J.W. discussed with A.G., X.W. and F.W. for the analysis and discussion of results. A.G. and J.W. were mainly responsible for preparing the manuscript with further inputs from other authors. All the authors discussed the results and commented on the manuscript.

## Supplementary Material

Supplementary Information Supplementary Material

## Figures and Tables

**Figure 1:**
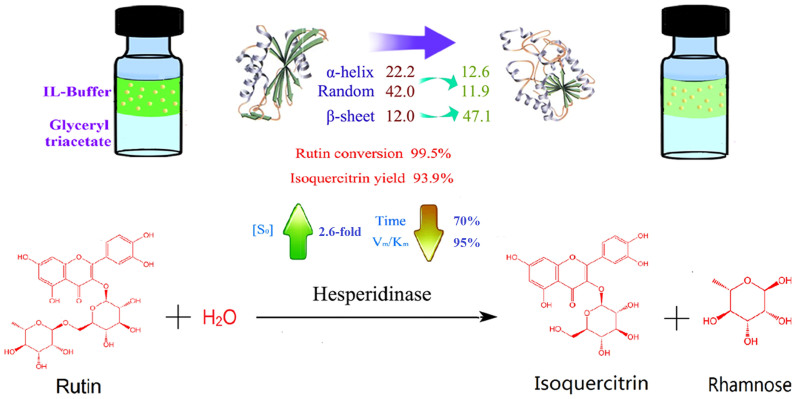
Biosynthesis of isoquercitrin using selective conversion of rutin catalyzed by hesperidinase.

**Table 1:** Comparative results of the enzymatic conversion of rutin to isoquercitrin in [Bmim][BF_4_]: glycine-sodium hydroxide (pH 9) (10:90,v/v)/glyceryl triacetate (1:1,v/v) and [Bmim][BF_4_]: glycine-sodium hydroxide (pH 9) (10:90,v/v) systems

Medium	Temperature of reaction (°C)	Substrate concentration (mmol/L)	Enzyme concentration (U/L)	Reaction time (h)	*V*_m_/*K*_m_ (h^−1^)	Rutin conversion (%)	Isoquercitrin yield (%)
[Bmim][BF_4_]:glycine-sodium hydroxide (pH 9) (10:90,v/v)	40	1.3	100	10	1531.4	93.4	91.4
[Bmim][BF_4_]:glycine-sodium hydroxide (pH 9) (10:90,v/v)/glyceryl triacetate (1:1,v/v)	45	3.3	10	3	6.9	99.5	93.9

**Table 2:** Hesperidinase secondary structure data in different systems

Medium	*α*-helix	*β-*sheet	Turn	Random	Total
Pure water	22.2	12.0	23.8	42.0	100
[Bmim][BF_4_]:glycine-sodium hydroxide (pH 9) (10:90,v/v)^a^	15.7	0	69.1	15.2	100
[Bmim][BF_4_]:glycine-sodium hydroxide (pH 9) (10:90,v/v)/glyceryl triacetate (1:1,v/v)^b^	12.6	47.1	28.4	11.9	100

^a^Rutin solution concentration, 3.3 mmol/L; reaction temperature, 45°C; hesperidinase concentration, 0.18 U/L.

^b^Rutin solution concentration, 3.3 mmol/L; reaction temperature, 45°C; hesperidinase concentration, 0.18 U/L.
