# Supplementary material for: An effective biphase system accelerates hesperidinase-catalyzed conversion of rutin to isoquercitrin
Source: Sci Rep. 2015 Mar 3;5:8682. doi: 10.1038/srep08682 (PMC4346833; doi:10.1038/srep08682)
Supplement: Supplementary Information — Supplementary Material [file srep08682-s1.doc]

**Supplementary materials for**

**An effective biphase system accelerates hesperidinase-catalyzed conversion of rutin to isoquercitrin**

Jun Wang, An Gong, Cai-Feng Yang, Qi Bao, Xin-Yi Shi, Bei-Bei Han, Xiang-Yang Wu, Fu-An Wu

**HPLC chromatogram of rutin and isoquercitrin by adding hesperidinase in reaction system**

Figs. S1A and S1B show the hydrolysis sample HPLC chromatogram using rutin as a substrate in the optimized biphase system. Rutin has a large peak area before the addition of hesperidinase and isoquercitrin has a large peak area after the addition of hesperidinase^1^. Fig. S1A reveals the liquid phase diagram of enzymatic synthesis before hesperidinase addition, Fig. S1B reveals the liquid phase diagram of enzymatic synthesis after hesperidinase addition.


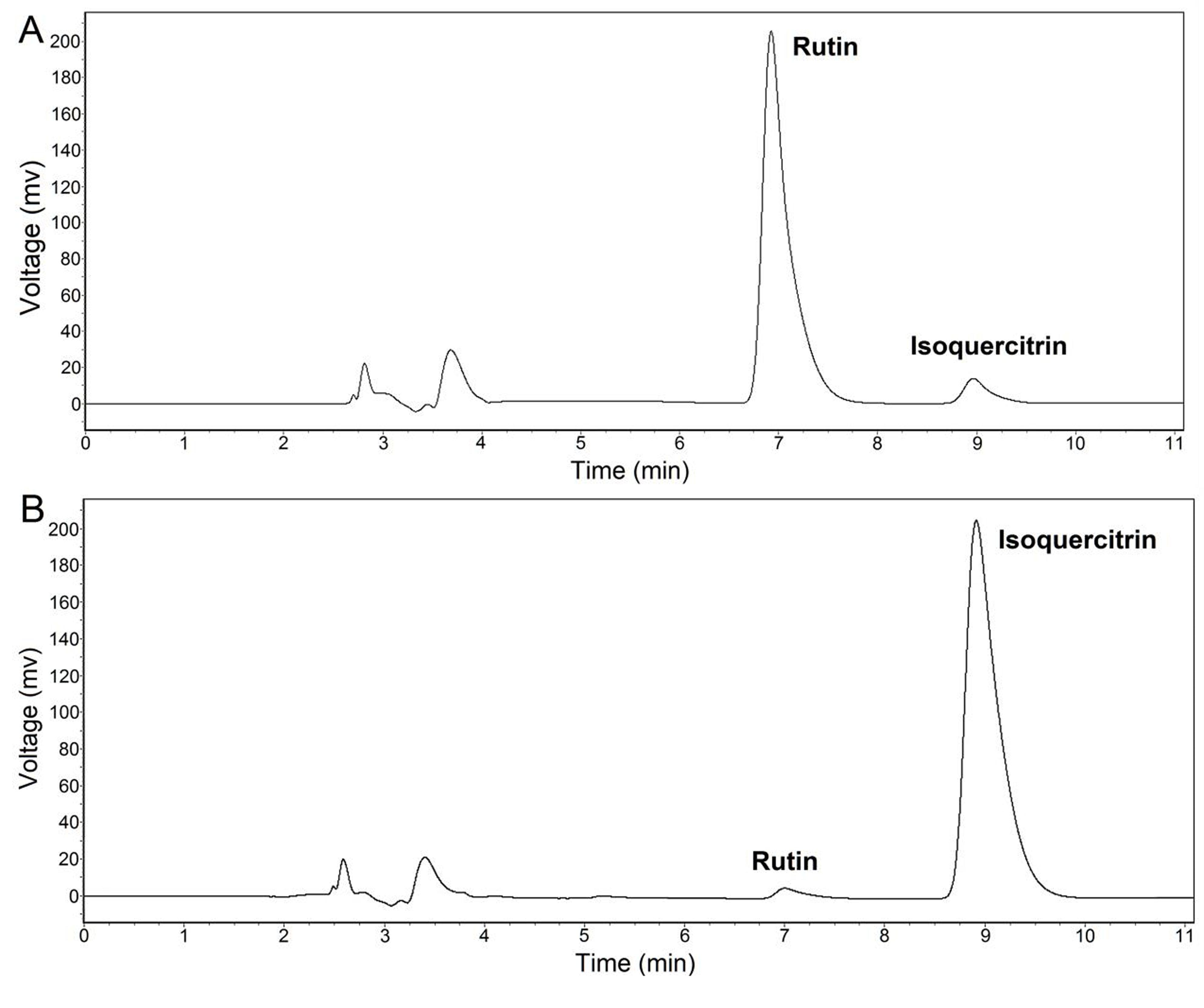


Fig.S1 The HPLC chromatogram of enzymatic synthesis of isoquercitrin from rutin in a biphase system. (A) The liquid phase diagram of enzymatic synthesis before hesperidinase addition; (B) The liquid phase diagram of enzymatic synthesis after hesperidinase addition.

**Calculation of** **partition coefficient in a biphase system.** All studies were performed using high-quality chemicals. Glyceryl triacetate was used as the extraction agent in a biphase system. Focusing on the glyceryl triacetate–buffer solutions partition coefficient for the substances of rutin and isoquercitrin, this quantity relates to the equilibrium between the substances in the mutually saturated media, which for simplicity may be designated by the solubility of the substance in the extraction agent.

The reaction systems, contained 720 *µ*L 3.3mmol/L rutin solution (pH=9 glycine-sodium hydroxide buffer solution), 100 *µ*L [Bmim][BF_4_], 180 *µ*L 10 U/L hesperidinase solution and 1 mL glyceryl triacetate, were performed in a temperature (45°C) controlled incubator shaker for 6 h. Take the average of three parallels of rutin and isoquercitrin concentrations. Rutin concentrations were 8.5×10^-2^ mmol/L and 2.1×10^-2^ mmol/L in aqueous phase and organic phase, respectively. Isoquercitrin concentrations were 1.1 mmol/L and 1.8 mmol/L in aqueous phase and organic phase, respectively.

And the partition coefficient is given by:

$m = \frac{c_{organic phase}}{c_{aqueous phase}}$ (S1)

where *c*_organic phase_ represents the concentration of the substance in organic phase, *c*_aqueus phase_ represents the concentration of the substance in aqueous phase.

The form of Eq. (S1) suggests that partition coefficient (m) is dimensionless, and it is generally taken to be so^2^. Partition coefficients of rutin and isoquercitrin are 0.25 and 1.6 in the two-phase system respectively. The extraction efficiency can be indicated by the separation factor^2^. The separation factor can be calculated by:

$\text{Separation factor（}\beta\text{）=}\frac{D_{isoquercitrin}}{D_{rutin}}$ (S2)

Although the value of partition coefficients of rutin (*D*_rutin_=0.25) and isoquercitrin (*D*_isoquercitrin_=1.6) were small, the separation factor *β* is 6.4. The larger the separation factor *β*, the better the extraction effects^3^. So, isoquercitrin could be better extracted from the mixed system.

Thus, isoquercitrin could be well separated from the aqueous phase to glyceryl triacetate, and rutin almost all remains in the buffer solution.

**References**

1. Wang, J., Sun, G., Yu, L., Wu, F. & Guo, X. Enhancement of the selective enzymatic biotransformation of rutin to isoquercitrin using an ionic liquid as a co-solvent. *Bioresource Technol.* **128**, 156-163 (2013).
2. Jimenez-Morales, I. et al. Production of 5-hydroxymethylfurfural from glucose using aluminium doped MCM-41 silica as acid catalyst. Appl. Catal. B-Environ. **164,** 70-76 (2015)
3. Baheri, B. et al. Performance of PVA/NaA Mixed Matrix Membrane for Removal of Water from Ethylene Glycol Solutions by Pervaporation. Chem. Eng. Commun. **202,** 316-321 (2015)
